# Impact of different approaches to calculation of treatment activities on achieved doses in radioiodine therapy of benign thyroid diseases

**DOI:** 10.1186/s40658-018-0231-x

**Published:** 2018-12-12

**Authors:** Jochen Hammes, Lutz van Heek, Melanie Hohberg, Manuel Reifegerst, Simone Stockter, Markus Dietlein, Markus Wild, Alexander Drzezga, Matthias Schmidt, Carsten Kobe

**Affiliations:** Department of Nuclear Medicine, University Hospital of Cologne, Kerpener Str. 62, 50937 Cologne, Germany

**Keywords:** Benign thyroid disease, Radioiodine therapy

## Abstract

**Purpose:**

Radioiodine has been used for the treatment of benign thyroid diseases for over 70 years. However, internationally, there is no common standard for pretherapeutic dosimetry to optimally define the individual therapy activity. Here, we analyze how absorbed tissue doses are influenced by different approaches to pretherapeutic activity calculation of varying complexity.

**Methods:**

Pretherapeutic determination of treatment activity was retrospectively recalculated in 666 patients who had undergone radioiodine therapy for benign thyroid diseases (Graves’ disease, non-toxic goiter, and uni- and multinodular goiter). Approaches considering none, some, or all of a set of individual factors, including target volume, maximum radioiodine uptake, and effective half-life, were applied. Assuming individually stable radioiodine kinetics, which had been monitored twice a day under therapy, hypothetically achieved tissue doses based on hypothetically administered activities resulting from the different methods of activity calculation were compared to intended target doses.

**Results:**

The Marinelli formula yields the smallest deviations of hypothetically achieved doses from intended target doses. Approaches taking individual target volume into consideration perform better than fixed therapy activities, which lead to high variances in achieved doses and high deviations of hypothetically achieved doses from intended target doses.

**Conclusion:**

Elaborate pretherapeutic dose planning, taking individual radioiodine uptake, half-life, and target volume into consideration, should be used whenever possible. The use of disease-specific fixed activities cannot be recommended. Deviations of achieved tissue doses from target doses can already be significantly lowered by application of volume-adapted treatment activities if more elaborate means are not available.

## Introduction

Radioiodine has been used for the treatment of benign thyroid diseases for over 70 years [1]. It is regarded as a safe method for the treatment of hyperthyroidism, and it can be used for volume reduction of enlarged thyroid glands (non-toxic goiter) [2] with only a low probability of side-effects [3].

The European Council Directive 97/43/Euratom [4] requires that for all medical exposure of individuals for radiotherapeutic purposes, exposures of target volumes shall be individually planned, and stresses that the doses of non-target volumes be as low as reasonably achievable. However, both inside and outside Europe, the different approaches to dosimetric calculations are the subject of lively debate [5, 6]. Some authors prefer to use fixed diagnosis-dependent activities, while others favor an individually planned dose concept [5–7]. The European Association of Nuclear Medicine and the Society of Nuclear Medicine both recommend uptake measurements but also leave room for disease-specific fixed activities [8, 9], while on the other hand, the German guideline strongly encourages more elaborate measures to determine the individual treatment activity [7].

Target doses specific to the disease entity are well established for Graves’ disease [10–12], non-toxic goiter [2], and toxic multinodular [13, 14] and toxic uninodular goiter [15]. Several methods exist for calculation of the activity to be administered, each aiming to achieve a predefined dose to the thyroid gland appropriate for the treatment of that specific thyroid disease [16]. Variables that need to be determined in a pretherapeutic radioiodine test include thyroid volume, effective radioiodine half-life in the thyroid gland, and thyroidal radioiodine uptake.

The achieved doses to the thyroid may vary depending on the method selected for activity calculation. The following approaches could be used in theory:

1. Fixed activities for each disease, independent of individually determined parameters
2. Standard activities for individual target volumes, independent of individual radioiodine uptake and individual radioiodine half-life (T1/2)
3. Individually calculated activities, taking into account individual target volume and individually determined radioiodine uptake but using literature values for disease-specific T1/2
4. Individually calculated activities, taking into account individual target volume and individual T1/2 but using literature values for radioiodine uptake
5. Individually calculated activities, taking into account individual values for target volume, radioiodine uptake, and T1/2 according to EANM SOP for activity calculation [16]

Our aim was to retrospectively analyze how the doses achieved in the thyroid would have been influenced by alternative methods of activity calculation, as listed above, in a cohort of patients who had undergone radioiodine therapy. We retrospectively estimated the hypothetically achieved dose in 666 patients with benign thyroid diseases, based on individual effective radioiodine uptake during therapy, thyroid volume, and effective half-life determined during therapy, for each method of pretherapeutic activity calculation.

## Materials and methods

### Patients

A total of 697 patients were treated with radioiodine therapy for benign thyroid disease between 25 May 2011 and 12 September 2012, in the Department for Nuclear Medicine at the University Hospital of Cologne.

Inclusion criteria for this analysis were:

- Diagnosis of Graves’ disease, non-toxic goiter, toxic multinodular goiter, or toxic uninodular goiter
- Pretherapeutic radioiodine test with radioiodine uptake measurements after 24 h, again after 5 days, and additionally after 6 h for Graves’ disease
- Withdrawal of thyreostatic drugs 2 days before the pretherapeutic radioiodine test until 1 day after application of the radioiodine test activity
- Withdrawal of thyreostatic drugs 2 days before radioiodine therapy
- Compensated metabolic state
- Serial peritherapeutic radioiodine uptake measurements twice a day for the duration of hospitalization

A total of 666 patients fulfilled the inclusion criteria.

### Thyroid volume calculation

The thyroid volume was determined before therapy by ultrasound (Siemens Sonoline Prima, 7.5 MHz). The thyroid volume was approximated by assuming ellipsoidal shape of both lobes and measuring three orthogonal diameters. The volume of one lobe was calculated as follows:

$$ {\mathrm{Volume}}_{\mathrm{lobe}}=\mathrm{length}\times \mathrm{thickness}\times \mathrm{width}\times \frac{1}{2} $$

Total thyroidal volume was considered as the target volume in Graves’ disease, non-toxic goiter, and toxic multinodular goiter, while in toxic uninodular goiter, the target volume was taken as equal to the adenomal volume. A tissue density of 1 g/cm^3^ was assumed.

### Radioiodine testing and therapy

The method of pretherapeutic radioiodine test and posttherapy dosimetric measurements has been published previously [16, 17]. The pretherapeutic radioiodine test was performed 10 to 14 days before radioiodine therapy. An average activity of 2.5 MBq ^131^I was applied orally. Radioiodine uptake measurements were performed after 24 h and again after 5 days. In Graves’ disease, an additional uptake measurement was performed 6 h after application. Thyreostatic drugs such as thiamazole, carbimazole, or propylthiouracile were paused from 2 days before until 1 day after radioiodine application.

The effective half-life was calculated according to:

$$ T1/{2}_e=\frac{\ln (2)\times \left({t}_2-{t}_1\right)}{\ln \left({\mathrm{RIU}}_1\right)-\ln \left({\mathrm{RIU}}_2\right)} $$

where RIU_*t*_ is the radioiodine uptake in percent at time point *t* = *t*_1_ or *t*_2_ and *T*1/2_*e*_ is the effective half-life during radioiodine test [days].

Activity calculation was performed according to Hänscheid et al. [16] with an intended target dose of 250 Gy for Graves’ disease, 150 Gy for non-toxic goiter and toxic multinodular goiter, and 400 Gy for toxic uninodular goiter. Radioiodine therapy was performed according to German regulations for radiation protection in relation to in-patient treatments. Uptake measurements were performed every 12 h after radioiodine application until discharge and the effectively achieved doses estimated.

### Calculation of hypothetically achieved doses

#### Fixed therapy activities

Ratios between target dose and effectively achieved doses for each patient were calculated and used as correction factors to determine the optimized individual activity, which if applied would have led to an effectively achieved dose equal to the target dose. Disease entity-specific fixed optimized therapy activities (*A*_opt,1_) were calculated as averages of individual *A*_opt_.

*D*_ha,1_ for fixed activities were determined by individually correcting *D*_*a*_ by the ratio of *A*_opt,1_ and the activity that had actually been applied *A*_ind_.

$$ {D}_{\mathrm{ha},1}={D}_a\times \frac{A_{\mathrm{opt},1}}{A_{\mathrm{ind}}} $$

where *D*_*a*_ is the effectively achieved dose, *D*_ha_ is the hypothetically achieved dose, *A*_opt,1_ is the disease entity-specific fixed therapy activities, and *A*_ind_ is the activity actually applied in the respective individual.

#### Standard activities for individual target volumes

Individual optimized therapy activities per milliliter target volume (*A*_opt,2_) were determined and averaged for each disease entity. *D*_ha,2_ were determined as in Method 1, taking into account the individual target volume.

$$ {D}_{\mathrm{ha},2}={D}_{\mathrm{a}}\times \frac{A_{\mathrm{opt},2}}{A_{\mathrm{ind}}}\times {V}_{\mathrm{T}} $$

where *V*_*t*_ is the target volume and *D*_*a*_, *D*_ha_, *A*_opt,2_, and *A*_ind_ as described above.

#### Calculated activities with individual target volume and individual radioiodine uptake

Individual optimized therapy activities per milliliter target volume with respect to individual maximum radioiodine uptake during the pretherapeutic radioiodine test (*A*_opt,3_) were determined and averaged for each disease entity. *D*_ha,3_ were calculated as in Methods 1 and 2, also taking into account individual maximum radioiodine uptake during the pretherapeutic radioiodine test.

$$ {D}_{\mathrm{ha},3}={D}_{\mathrm{a}}\times \frac{A_{\mathrm{opt},3}}{A_{\mathrm{ind}}}\times \frac{V_T}{\mathrm{RIU}} $$

where RIU is the individual radioiodine uptake as determined during the pretherapeutic radioiodine test and *V*_t_, *D*_*a*_, *D*_ha_, *A*_opt,3_, and *A*_ind_ as described above.

#### Calculated activities with individual target volume and individual T1/2

Individual optimized therapy activities per milliliter target volume with respect to individual T1/2 during the pretherapeutic radioiodine test (*A*_opt,4_) were determined and averaged for each disease entity. *D*_ha,4_ were calculated as in Methods 1 and 2, also taking into account individual T1/2 during the pretherapeutic radioiodine test.

$$ {D}_{\mathrm{ha},4}={D}_a\times \frac{A_{\mathrm{opt},4}}{A_{\mathrm{ind}}}\times \frac{V_T}{T1/2} $$

where *T*1/2 is the individual effective radioiodine half-life as determined during the pretherapeutic radioiodine test and *V*_*t*_, *D*_a_, *D*_ha_, *A*_opt,4_, and *A*_ind_ as described above.

### Activities calculated according to EANM SOP

Methods of calculation 1–4 were compared to established methods, i.e., the formulas of Marinelli (SOP equation 8) and Bockisch (SOP equation 9), described in EANM Standard Operational Procedures for Dosimetry prior to radioiodine therapy of benign thyroid diseases [14]. Doses that could have been achieved in patients were calculated based on hypothetically administered activities derived using the EANM-recommended procedures of Marinelli and Bockisch in addition to the approaches 1–4.

### Deviations of hypothetically achieved doses from the target dose

The root mean squared differences between the individual target dose (*D*_*t*_) and the individual hypothetically achieved dose (*D*_ha,i_) were calculated for each disease entity and for each method of calculation according to:

$$ {\Delta }_i=\sqrt{\frac{1}{n}\sum {\left({D}_t-{D}_{\mathrm{ha}}\right)}^2} $$

where *Δ*_*i*_ is the root mean squared difference between *D*_ha,I_ and *D*_*t*_, *n* is the number of patients for each disease entity, and *i* is the method of calculation.

Root mean squared deviations of *D*_ha_ from the target dose for hypothetically administered activities determined based on the formulas of Bockisch and Marinelli were calculated in the same way. All possible pairs of *Δ*_*i*_ were then compared for each disease entity via paired *t* test. *P* levels below 0.05 were considered significant.

## Results

Patient characteristics are listed in Table 1. Group-wise optimized activities to reach the intended target dose (*A*_opt,i_) for calculations based on approaches 1–4 are listed in Table 2. It is of note that using all volume-adapted calculation approaches (i.e., approaches 2–4) resulted in significantly higher optimized activities for toxic uninodular goiter than for Graves’ disease, toxic multinodular goiter, and non-toxic goiter. This may be explained in part by the relatively high target dose per milliliter target volume in toxic uninodular goiter. However, the activity ratio exceeds the target dose ratio.

**Table 1:** Patient characteristics

	GD	NTG	TMG	TUG
Number of patients	206	74	279	107
Age (years) ± SD	48 ± 16	64 ± 13	66 ± 12	62 ± 13
Age range	17–86	36–86	23–82	32–85
Sex (male/female)	25/181	22/52	70/209	29/78
Thyroid mass (g) ± SD	25 ± 16	73 ± 47	45 ± 37	8 ± 6

*GD* Graves’ disease, *NTG* non-toxic nodular goiter, *TMG* toxic multinodular goiter, *TUG* toxic uninodular goiter, *SD* standard deviation

**Table 2:** Optimized activities (*A*_opt,i_) determined in approaches 1–4 to calculation

Activity calculation approach	TUG	NTG	TMG	GD	Unit
1	540 (477)	1229 (1342)	627 (505)	578 (882)	MBq
2	69 (41)	16 (10)	15 (10)	24 (26)	MBq g^−1^
3	1898 (1010)	509 (189)	513 (181)	1424 (1328)	MBq % g^−1^
4	429 (229)	120 (62)	111 (72)	117 (97)	MBq d g^−1^

Standard deviations are given in brackets. The ratio between optimized activities of TUG and the other disease entities far exceeds the ratio between target doses

*GD* Graves’ disease, *NTG* non-toxic nodular goiter, *TMG* toxic multinodular goiter, *TUG* toxic uninodular goiter

Hypothetically achieved doses for treatment activities derived from approaches 1–4 and activity calculations based on the EANM SOP equations 8 and 9 of Marinelli and Bockisch respectively are listed in Table 3. For all disease entities, the variance in *D*_ha_ is highest for approach 1 with fixed activities reflecting the lack of consideration of patient-individual factors. For toxic multinodular goiter and non-toxic goiter, all approaches taking at least individual thyroid volume into account yield fairly low variances in all disease categories, while in Graves’ disease, an activity calculation based on EANM SOP results in the lowest variance for any disease entity.

**Table 3:** Hypothetically achieved doses (*D*_ha_) for approaches 1–4 and activities derived from the EANM equations SOP 8 and 9 of Marinelli and Bockisch, respectively

	TUG	NTG	TMG	GD
D_ha,1_	748 (708)	291 (285)	234 (177)	403 (215)
D_ha,2_	518 (251)	182 (70)	179 (64)	338 (129)
D_ha,3_	489 (196)	165 (49)	167 (72)	313 (114)
D_ha,4_	499 (244)	182 (76)	179 (68)	310 (110)
D_ha,Marinelli_	370 (120)	149 (39)	161 (71)	251 (58)
D_ha,Bockisch_	379 (127)	159 (43)	170 (75)	251 (60)

Standard deviations are given in brackets. Standard deviations are highest for calculations performed by approach 1 with fixed activities reflecting the lack of consideration of patient-individual factors

*GD* Graves’ disease, *NTG* non-toxic nodular goiter, *TMG* toxic multinodular goiter, *TUG* toxic uninodular goiter

**Fig. 1:**
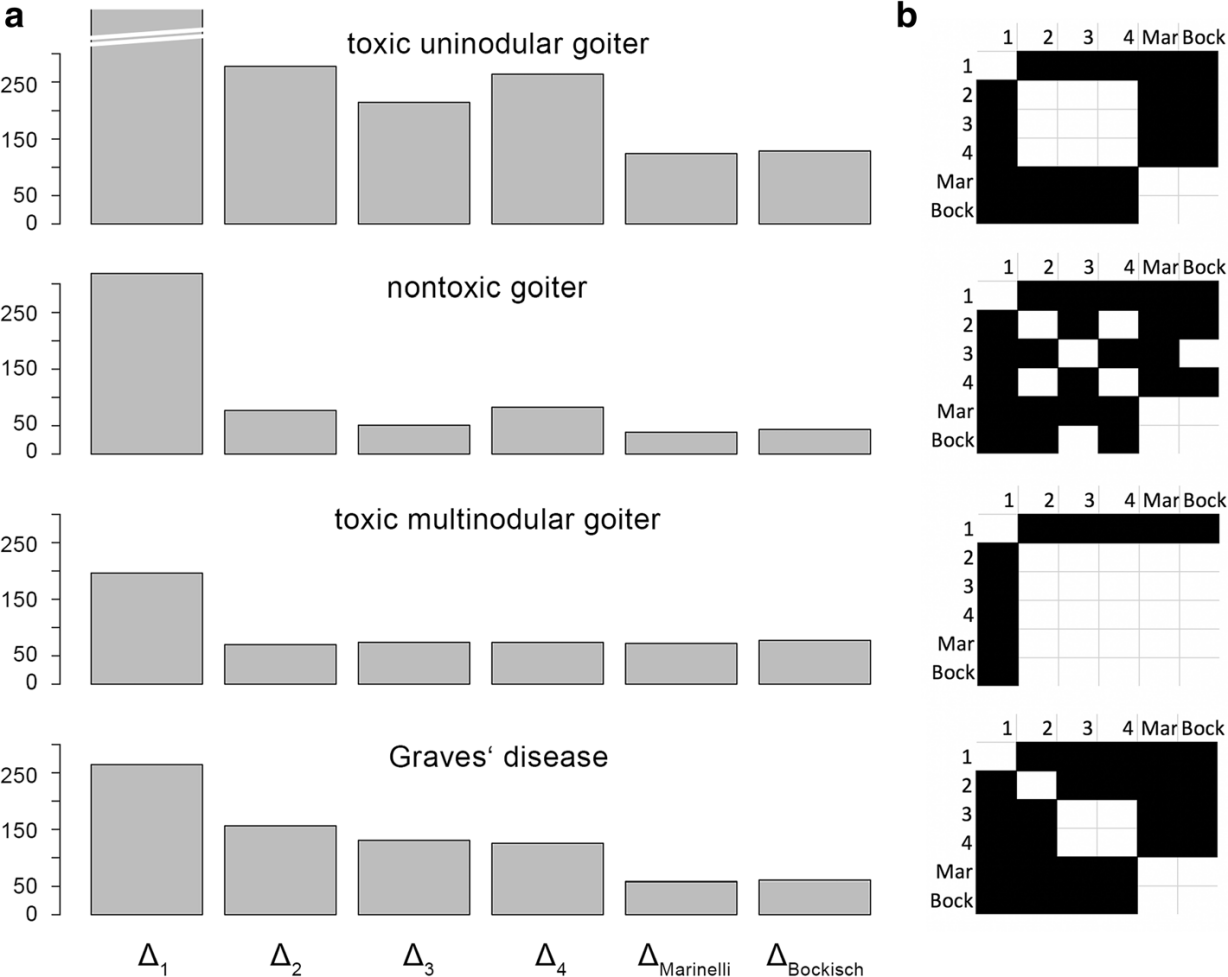
Disease entity-wise root mean squared deviations *Δ*_*i*_ of hypothetically achieved doses. **a** Root mean squared deviations *Δ*_*i*_ of hypothetically achieved doses (*D*_ha_) in Gy from target doses (*D*_*t*_) for approaches 1–4 and activities derived from EANM SOP 8 (Marinelli) and 9 (Bockisch) for each disease entity. **b** Results of pair-wise *t* tests between root mean squared deviation comparing all approaches. Black cells indicate significant differences between compared groups (i.e., *p* < 0.05)

The root mean squared deviations of the hypothetically achieved doses from the target doses are shown in Table 4 and Fig. 1a. None of the simplified approaches to calculation resulted in root mean squared deviations of hypothetically achieved doses *D*_ha_ from target doses *D*_*t*_ that were smaller than those of current EANM SOP. Approach 1 with fixed activities not taking any individual factors into account results in the highest deviations between *D*_t_ dose and *D*_ha_ dose across all disease entities (*p* < 0.05 for all disease entities). Approaches 2–4 result in lower dose deviations in all disease entities. However, in non-toxic goiter, Graves’ disease, and toxic unimodular goiter, dose deviations based on EANM SOP approaches resulted in significantly lower dose deviations than all of the simplified approaches.

**Table 4:** Root mean squared deviations *Δ*_*i*_ of *D*_ha_ from *D*_*t*_ (MBq) for approaches 1–4 and activities derived from the EANM equations SOP 8 and 9 of Marinelli and Bockisch, respectively

	TUG	NTG	TMG	GD
Δ_1_	789	318	196	264
Δ_2_	278	77	70	156
Δ_3_	215	51	74	131
Δ_4_	264	83	74	125
Δ_Marinelli_	124	39	72	58
Δ _Bockisch_	129	43	77	61

Smallest deviations of *D*_ha_ from *D*_*t*_ were found for activity calculations based on the current EANM SOP across all disease entities. Results of pair-wise *t* tests are displayed in Fig. 1b

*GD* Graves’ disease, *NTG* non-toxic nodular goiter, *TMG* toxic multinodular goiter, *TUG* toxic uninodular goiter

Results of the statistical comparison of root mean squared deviations of *D*_ha_ from *D*_*t*_ for every possible pair of activity calculations are depicted in matrix form in Fig. 1b. Black cells indicate statistically significant differences. In toxic multinodular goiter, there is no statistically significant difference between the deviations of *D*_ha_ from *D*_*t*_ resulting from approaches 2–4 and those resulting from EANM SOPs. In Graves’ disease, approaches 2–4 perform better than a fixed activity concept, but lowest deviations of *D*_ha_ from *D*_*t*_ are achieved by conventional activity calculation according to EANM SOP.

## Discussion

The following findings emerge from our analysis of different approaches to activity calculation in 666 patients treated with radioiodine for benign thyroid disease:

(i) In general, an elaborate dosimetric approach following the European SOP [16] yields the most accurate results with smallest deviations of hypothetically achieved doses from intended target doses.
(ii) The Marinelli formula remains the most accurate means of dose calculation in toxic multinodular goiter. However, all other individualized approaches taking at least the individual thyroid volume into account result in relatively low deviations of hypothetically achieved doses from target doses.
(iii) Fixed activities lead to high variances in achieved doses and high deviations of hypothetically achieved doses from target doses.

The general aim of radioiodine treatment is to eliminate hyperthyroidism and to shrink an enlarged goiter. Here, activity calculation based on the EANM SOP formulas offers lowest deviations of achieved doses from target doses. Some investigators raise concern about the lack of evidence of the benefits of individualized dosimetry for radioiodine therapy in terms of cure rates [5, 6]. In fact, there is some controversy over dosimetric approaches in general.

Accurate dosimetry requires specialized knowledge and experience, which is not available in all clinical centers. Hence, fixed activities are still used in nuclear medical treatment—not only in malignant diseases [18, 19].

A dose dependency of success rates in the treatment of hyperthyroidism was shown back in 1967 in a study by Smith and Wilson [20]. Leslie et al. prospectively compared outcome in 88 patients with Graves’ disease, using fixed and adjusted activities [21]. Adjusted activities respected individual uptake and thyroid volume as estimated clinically without ultrasound. Hyperthyroidism was eliminated in 34 out of 43 patients (79%) with adjusted activities and in 33 out of 45 patients (73%) treated with fixed activities. Due to the low number of patients, the authors could not report any significant differences between fixed and individualized treatment activities. In a further prospective randomized trial, Jaiswal et al. [5] had observed success rates for the treatment of Graves’ disease of 65% for individualized activity calculation as compared to 60% for fixed activities. Here again, individual activity calculations resulted in a numerically higher success rate, but due to the low number of patients, the authors could not produce evidence of a significant difference between methods.

Alexander and Larsen retrospectively analyzed patients who were treated after a dosimetric approach restricted to thyroidal uptake measurements. They found a direct relation of treatment success to the absorbed dose to thyroid tissue in a retrospective analysis of 261 patients [22]. The relation between the absorbed dose and success rate was confirmed by Peters et al. and Boelaert et al. [23, 24], indicating that pretherapeutic activity calculation is worthwhile.

Elimination of hyperthyroidism was observed in 67% of GD’s patients in a study by Catargi et al. with a target dose of only 50 Gy, using a dosimetric approach with individual sonographic determination of thyroidal volume and estimation of uptake and half-life [25]. When comparing size-adapted fixed doses with an activity calculation based on uptake and volume measured by ultrasound, Jarlov and colleagues found no significant difference in 163 patients [26]. Here, one might argue that volume adaption is wholly sufficient as compared to a full dosimetric approach. And in fact, we found in this study that all volume-adapted strategies are superior to fixed doses in terms of accuracy. As 62 out of 163 patients who were treated by Jarlov and co-workers [26] remained hyperthyroid, the overall success rate was no higher than 62%. Today, success rates of over 90% are possible when higher absorbed doses are intended, as in Graves’ disease where hypothyroidism is the intended treatment outcome [27]. To ensure such high success rates, fixed treatment activities would be needed, which in some cases could far exceed the activity needed for sufficient treatment [28], and would in turn lead to relevant unnecessary radiation exposure.

As nuclear medicine physicians, our aim is to keep radiation exposure as low as reasonably possible. We would like to point out that we do not consider a mere reduction of disease-specific fixed doses a valid approach to reduce radiation exposure as this would lead to a significant amount of therapy failures due to the high variance of the resulting target doses. But we understand that an accurate pretherapeutic activity calculation is not available in all countries for all patients undergoing radioiodine treatment for benign thyroid diseases. We further acknowledge that economic circumstances may prevent installation of radioiodine testing facilities and preclude the availability of a medical physicist. Implementation of dosimetry has substantial implications for infrastructure resourcing. Bearing in mind that we are in an era when personalized treatments are the focus of many medical disciplines and acknowledging the marked increase in availability of ultrasound volume estimation today, it is difficult to justify not applying at least a volume-adapted dose concept when treating patients with hyperthyroidism and aiming to achieve hypothyroidism.

Especially in toxic multinodular goiter, we have learned that higher target doses frequently lead to hypothyroidism which is generally not intended [13] and requires more complex post-therapeutic patient management due to the need for daily medication. Encouraging data have shown that individualized dose concepts may avoid hypothyroidism in toxic multinodular goiter in more than 90% of cases [29].

Target doses between 100 and 150 Gy have been recommended for radioiodine therapy of toxic multinodular goiter [7, 8]. Here, variable target doses adapted to levels of severity of hyperthyroidism may offer more individualized and risk-adapted therapy strategies. Higher doses, e.g. 120 Gy, might safely eliminate hyperthyroidism in cases where TSH-levels lie below 0.2 mU/l while lower target doses (e.g. 100 Gy) could be sufficient for TSH-levels of 0.2–0.4 mU/l. Thus, an accurate and robust system of pretherapeutic dosimetry appears to be indispensable.

Based on our data, we recommend pretherapeutic activity calculation for benign thyroid diseases, as described in the European guidelines [16]. Considering the data on dose-effect relations, we advise against the use of fixed activity concepts.
